# 858. Early Fresh Frozen Plasma Use in Pediatric Burn Resuscitation: A Retrospective Analysis

**DOI:** 10.1093/jbcr/irag033.328

**Published:** 2026-04-06

**Authors:** Kelsey G Glover, Rohit Mittal, Adria Johnson, Aaron P Lesher, Steven A Kahn

**Affiliations:** Medical University of South Carolina, Charleston, SC; South Carolina Burn Center at Medical University of South Carolina, Charleston, SC; Medical University of South Carolina, Charleston, SC; Medical University of South Carolina, Charleston, SC; Medical University of South Carolina, Charleston, SC

## Abstract

**Introduction:**

Appropriate fluid resuscitation is critical in pediatric burn patients to prevent consequences of burn shock and maintain adequate tissue perfusion. Under-resuscitation is not the only issue that may arise with fluid management; over-resuscitation is equally problematic. The pediatric population is prone to “fluid creep” from multiple sources, which include pre-hospital fluid administration, hidden, and resuscitative fluids. The authors recently published their experience using early FFP to decrease resuscitative volumes and improve outcomes in adults. We hypothesize that plasma administration is safe and feasible in the pediatric burn population.

**Methods:**

This retrospective study (2023-2025) examined pediatric burn patients (>20% TBSA) resuscitated with early FFP administration and rescue FFP for oliguria. Patients <13 years received crystalloid at 3 cc/kg/%TBSA plus 10 cc/kg FFP for 30-50% TBSA burns or 20 cc/kg FFP for >50% TBSA burns (goal UOP 1 cc/kg/hr). Patients >13 years received 2 cc/kg/%TBSA using adjusted body weight plus 1 unit FFP for 30-50% TBSA or 2 units FFP for >50% TBSA burns (goal UOP 0.5 cc/kg/hr). Demographics, injury characteristics, fluids administered, UOP, outcomes, and mortality were recorded.

**Results:**

Patients <13 years (n = 14, median age 5) with a mean TBSA of 32.5% received, on average, 4.25 cc/kg/%TBSA total fluid, including 15.08 cc/kg FFP, with a mean UOP of 1.68 cc/kg/hr. Patients >13 years (n = 3, median age 15) with a mean TBSA of 24.8% received 3.48 cc/kg/%TBSA total fluid and 1 unit FFP, with a mean UOP of 1.07 cc/kg/hr. When prehospital and hidden fluids were included, both groups exceeded 5 cc/kg/%TBSA. No mortality was observed. Eight patients required ventilator support (median 7 days). Outcomes included 1 tracheostomy, 3 escharotomies, and 2 mild transient acute kidney injuries (not requiring hemodialysis).

**Conclusions:**

Despite early FFP to reverse capillary leak, total fluid volume at 24 hours and subsequent UOP suggest patients received fluid volume in excess of their physiologic need. Our findings underscore the need for standardized reporting of fluid resuscitation efforts across institutions, as fluid creep can occur through multiple pathways, including prehospital administration, hidden, and formal resuscitative fluids. Comprehensive documentation of all fluid sources is crucial for an accurate assessment of resuscitation protocols and for meaningful inter-institutional comparison. The fluid resuscitation protocol has since been modified to include more substantial decreases in IV fluids when UOP is adequate. The absence of mortality and minimal adverse events supports the safety profile of FFP for early resuscitative use in pediatric burn patients.

**Applicability of Research to Practice:**

This study demonstrates that early FFP administration can be safely incorporated into pediatric burn resuscitation protocols, providing clinicians with an evidence based approach to mitigate fluid creep.

**Funding for the study:**

N/A.

